# Erectile dysfunction and quality of life in patients under left ventricular assist device support − an unspoken issue

**DOI:** 10.1016/j.ijcha.2023.101263

**Published:** 2023-08-22

**Authors:** Aiste Monika Jakstaite, Peter Luedike, Simon Wernhart, Markus Kamler, Arjang Ruhparwar, Tienush Rassaf, Maria Papathanasiou

**Affiliations:** a Department of Cardiology and Vascular Medicine, West German Heart and Vascular Center, University Hospital Essen, Hufelandstrasse 55, 45147 Essen, Germany; b Department of Thoracic- and Cardiovascular Surgery, West German Heart and Vascular Center, University Hospital Essen, Hufelandstrasse 55, 45147 Essen, Germany

**Keywords:** LVAD, Quality of life, Erectile function, Phosphodiesterase-5 inhibitors

## Abstract

**Background:**

Multiple domains of quality of life (QoL) such as erectile function are not sufficiently investigated among left ventricular assist-device (LVAD) patients. We aimed to evaluate the prevalence of erectile dysfunction (ED) and its association with QoL and depression.

**Methods:**

This is a prospective, single-center, cross-sectional study. We included adult male LVAD patients who were clinically stable after at least 3 months post-implantation. Erectile function was assessed with the International Index of Erectile Function (IIEF-5) questionnaire with a score of ≤21 being confirmatory for ED. QoL and depression were estimated with the Kansas City Cardiomyopathy Questionnaire (KCCQ) and the Patient Health Questionnaire depression scale (PHQ-8), respectively.

**Results:**

The study included 56 patients, of whom 45 (80 %) met criteria for ED, a prevalence much higher than previously reported in patients with established cardiovascular disease or conservatively treated heart failure. Patients with ED were older and had lower 6-minute walking distance. ED was not associated with comorbidities and heart failure medications but with less frequent use of diuretics and phosphodiesterase-5 inhibitors. There was a correlation between erectile function and depression as well as QoL.

**Conclusions:**

These findings underscore that ED deserves special attention and should be included in a multi-targeted approach to address suboptimal QoL outcomes after LVAD implantation.

## 1 Introduction

Left ventricular assist device (LVAD) therapy improves survival and quality of life (QoL) in selected patients with advanced heart failure (HF) [1], [2]. As device technology and complication management continue to improve, the number of patients on long-term support is growing and the need to achieve sustained benefits in QoL is becoming increasingly important. Recent reports demonstrated that a significant proportion of patients experience poor QoL after LVAD implantation [3], [4]. However, most studies relied on heart failure (HF) specific questionnaires to estimate QoL, which do not address multiple domains of QoL such as sexual function, mental health, and psychosocial factors. These aspects become more relevant as patients gain physical functioning and may be influenced by multiple lifestyle changes related to LVAD. Two studies have investigated sexual function among male and female LVAD recipients using a questionnaire that addresses pleasure, desire, arousal, and orgasm [5], [6] and reported that 70–80 % of patients met the criteria for sexual dysfunction. Erectile dysfunction (ED) is a highly prevalent condition, impairing QoL across a wide spectrum of HF patients. ED affects the vast majority of patients with advanced HF, with a reported prevalence reaching up to 96 % [7], [8]. Interestingly, studies on stable heart transplant recipients report that up to 71 % have some degree of ED, underscoring the multifactorial origin of ED among this patient collective [9], [10]. The issue of ED among male patients on LVAD support is still not adequately investigated. In this study, we aimed to analyze the prevalence of ED among LVAD recipients and its potential association with QoL and severity of depressive symptoms.

## 2 Methods

This prospective cross-sectional study included adult male patients on durable LVAD support. Patients were asked to participate in the study and completed the study questionnaires during their visits in the outpatient HF clinic. ED, defined as the inability to attain or maintain a penile erection sufficient for satisfactory sexual performance, was evaluated with the International Index of Erectile Function (IIEF-5) questionnaire [11]. According to IIEF-5 responses to five items are scored from 0 or 1 (worst) to 5 (best) and the final score ranges from 1 to 25 points with a descending score indicating worsening erectile function and a score ≤ 21 being confirmatory for ED. QoL was assessed with the Kansas City Cardiomyopathy Questionnaire (KCCQ) and depressive symptoms with the Patient Health Questionnaire depression scale 8 (PHQ-8) [12]. Informed consent was obtained from each patient. The study was approved by the institutional ethics committee (Registration-Nr: 22–10725-BO) and conforms to the ethical guidelines of the 1975 Declaration of Helsinki. The paired *t*-test and the Mann-Whitney *U* test were used for comparison of continuous data. Association between binary variables was estimated by the x^2^ or the likelihood ratio test. Pearson's correlation was run to assess the relationship between IIEF-5, KCCQ, and PHQ-8 scores.

## 3 Results

The study included 56 male patients on durable LVAD support. The mean age was 56 years and the median time on device support was 430 days (interquartile range, IQR: 147–1140). All patients were on continuous-flow LVAD support, with the vast majority (80 %) being implanted as a bridge to transplant. Clinical characteristics of the study patients are summarized in Table 1.

**Table 1:** Characteristics of the study patients.

Variable		Overall (n = 56)	ED (n = 45)	No ED (n = 11)	*p* value
Age, y		56 ± 12	58 ± 11	49 ± 11	0.022
HF etiology, n (%)	Ischemic Non-ischemic	29 (52) 27 (48)	23 (51) 22 (49)	6 (55) 5 (45)	0.838
Therapeutic goal, n (%)	Destination therapy Bridge to transplant	11 (20) 45 (80)	11 (24) 34 (76)	0 11 (100)	0.020
LVAD type, n (%)	HeartWare HVAD HeartMate 3	17 (30) 39 (70)	16 (36) 29 (64)	1 (9) 10 (91)	0.060
Duration of LVAD support, days*		430 (147–1140)	543 (154–1433)	209 (148–429.5)	0.103
NYHA class at enrollment, n (%)	I II III	7 (12.5) 35 (62.5) 14 (25)	5 (11) 29 (64) 11 (24)	2 (18) 6 (55) 3 (27)	0.542
Cardiopulmonary exercise testing	6MWD, m Peak oxygen uptake (ml/min/Kg) VE/VCO_2_ slope	367 ± 107 11.8 ± 4 33.7 ± 6.7	349 ± 108 10.9 ± 4 35.2 ± 6.2	428 ± 76 13.7 ± 3.3 30.3 ± 7	0.037 0.054 0.053
Medical therapy, n (%)	ARNI ACE-I/ARB MRA SGLT2i b-blocker Diuretics PDE5I Digitoxin CCB	27 (48) 21 (38) 48 (86) 19 (34) 53 (95) 47 (84) 29 (52) 6 (11) 8 (14)	23 (51) 16 (36) 37 (82) 14 (31) 42 (93) 36 (80) 20 (44) 5 (11) 7 (16)	4 (36) 5 (46) 11 (1 0 0) 5 (46) 11 (100) 11 (100) 9 (82) 1 (9) 1 (9)	0.380 0.547 0.051 0.375 0.245 0.037 0.026 0.843 0.565
	Antidepressants	8 (14)	6 (13)	2 (11)	0.649
Comorbidities, n (%)	Chronic kidney disease Diabetes COPD Arterial hypertension Atrial fibrillation Stroke Mental disorders	27 (48) 15 (27) 4 (7) 20 (36) 31 (55) 6 (11) 8 (14)	23 (51) 12 (27) 4 (9) 15 (33) 25 (56) 5 (11) 6 (13)	4 (36) 3 (27) 0 5 (46) 6 (55) 1 (9) 2 (11)	0.380 0.968 0.177 0.457 0.952 0.843 0.649
Complications on LVAD support, n (%)	Bleeding complications Infection HF decompensation Ventricular arrhythmias	13 (23) 11 (20) 4 (7) 24 (43)	10 (22) 9 (20) 4 (8.9) 19 (42.2)	3 (27.3) 2 (18.2) 0 5 (45.5)	0.754 0.891 0.177 0.846

ACE-I = angiotensin-converting enzyme inhibitor, ARB = angiotensin II receptor blocker, ARNI = angiotensin receptor neprilysin inhibitor, CCB = calcium Chanel Blockers, COPD = Chronic Obstructive Pulmonary Disease, ED = Erectile dysfunction, HF = heart failure, INTERMACS = Interagency Registry for Mechanically Assisted Circulatory Support, LVAD = left ventricular assist device, MRA = mineralocorticoid receptor antagonist, NYHA = New York Heart Association, PDE5I = phosphodiesterase type-5 inhibitor, SGLT2i = sodium‐glucose cotransporter 2 Inhibitor, 6MWD = 6-minute walking test distance.

* Values refer to median (interquartile range).

Based on the IIEF-5 score, 45 patients (80 %) met the criteria for ED. Median IIEF-5 score was 11 (IQR: 4.5–20.5). Patients with ED were older (58 vs. 49 years, p = 0.022). Despite an equal distribution of NYHA class, patients with ED exhibited worse exercise capacity with lower 6-minute walking distance (349 vs. 428 m, p = 0.037) and marginally lower peak oxygen uptake (11 vs. 14 ml/min/Kg, p = 0.054). Additionally, there was a weak correlation between IIEF-5 score and 6MWT (r = 0.47, p = 0.004), while no significant association was observed between peak oxygen uptake and IIEF-5 (r = −0.29, p = 0.058). There were no significant differences observed in the prevalence of ED and IIEF-5 scores between patients with HeartWare and HeartMate 3; the mean IIEF-5 scores were 12 vs. 8, p = 0.722, respectively. Both patient groups with and without ED exhibited similar characteristics in terms of HF etiology and LVAD support duration (Table 1). We found no association between ED and comorbidities or LVAD complications. ED was not associated with HF medications but less frequent use of diuretics (80 vs. 100 %, p = 0.037) and phosphodiesterase-5 inhibitors (PDE5I) (44 vs. 82 %, p = 0.026) were observed in patients with ED. PDE5I was used off-label to prevent right ventricular failure. None of the patients were under treatment for ED.

**Fig. 1:**
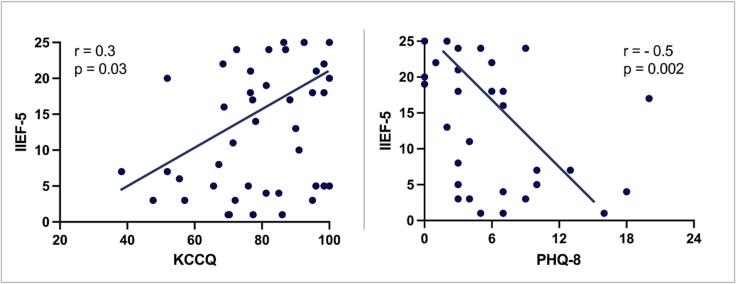
**Erectile function correlates with quality of life and severity of depressive symptoms.** IIEF-5 = International Index of Erectile Function, KCCQ = Kansas City Cardiomyopathy Questionnaire, PHQ-8 = Patient Health Questionnaire Depression Scale.

Based on the PHQ-8 score, 54 % met the criteria for depression, with a mean PHQ-8 score of 5 (IQR: 3–9), though only 14 % of the study patients had a known, medically managed mental disorder. Further analysis revealed the correlation between the IIEF-5 score and PHQ-8 score (r = -0.5, p = 0.002) and between IIEF-5 and KCCQ score (r = 0.33, p = 0.03) (Fig. 1).

## 4 Discussion

Despite the normalization of hemodynamics after LVAD, the prevalence of ED was high in this cohort of relatively young patients and even higher than previously reported in patients with established cardiovascular disease or conservatively treated HF [13]. A possible explanation is that ED is often of non-organic or multifactorial origin. Patients with ED were older and had shorter 6-minute walking distance but overall exhibited similar burden of comorbidities and HF medical treatment, as patients without ED. There was no association of ED with factors historically incriminated to be potentially causative agents, e.g. beta blockers, but diuretics and PDE5Is were more frequently prescribed in patients without ED. The latter is interesting since PDE5I remains the mainstay of therapy for ED but also due to the revived interest for these agents after LVAD implantation. PDE5Is have been associated with improved survival and lower rates of ischemic stroke in retrospective INTERMACS analyses, possibly due to their pleiotropic effects beyond pulmonary vasodilation [14].

In the Multicenter Study of MAGLEV Technology in Patients Undergoing Mechanical Circulatory Support Therapy with HeartMate 3 patients were considered to “live well with an LVAD” if they had achieved KCCQ score > 50 and good functional class (NYHA I/II or 6-minute walking distance > 300 m). At 6 months post-implantation, only 65 % of patients had achieved these goals [3]. Accordingly, better QoL outcomes after LVAD remain an unmet target that requires further attention and in-depth analysis of the various QoL components. Even if QoL was shown to be moderate to high in our cohort (mean KCCQ > 70 %), there was a correlation between erectile function and QoL as well as depressive symptoms. These findings underscore the interconnection of these domains and that holistic approaches that encompass multiple aspects of daily living may be more appropriate to assess QoL. Uncertainty persists regarding whether the restoration of cardiac output and organ perfusion via LVAD leads to improvements in sexual dysfunction, necessitating exploration of ED changes post LVAD implantation in future studies.

## 5 Study limitations

The study is limited by the small sample size which precludes adjustment for various variables and the lack of data before LVAD implantation for comparative analysis. However, this is one of the very few reports to touch upon an underrecognized issue in this growing patient cohort providing a comprehensive analysis of self-reported QoL measures.

## 6 Conclusions

PDE5I may be of potential benefit for ED. Their role should be further evaluated in future studies. The current findings underscore that ED is an important healthcare issue, and as such, it deserves special attention and should be included in a multi-targeted approach to address suboptimal QoL outcomes after LVAD implantation.

## Author contributions

AMJ, MP, PL and TR were responsible for the study design, data preparation, statistical analysis, and drafted the manuscript. SW, MK and AR drafted part of the manuscript and revised it for important intellectual content.

## Funding

This work was supported by the Universitätsmedizin Essen Clinician Scientist Academy (UMEA) and the German Research Foundation (DFG, Deutsche Forschungs‐Gemeinschaft) (Grant Nr: FU356/12‐1 to MP and AMJ) and the DFG (Grant Nr: RA969/12‐1 to TR).

## Declaration of Competing Interest

The authors declare that they have no known competing financial interests or personal relationships that could have appeared to influence the work reported in this paper.
